# Electrical impedance tomography-guided the optimal awake prone position in a moderate ARDS patient

**DOI:** 10.1186/s13054-025-05332-8

**Published:** 2025-03-03

**Authors:** Yongzhen Sun, Jiale Tao, Jinjun Jiang, Shujing Chen

**Affiliations:** 1 Department of Pulmonary and Critical Care Medicine, The Affiliated Hospital of ShaoXing University, Zhejiang, China; 2 Department of Pulmonary and Critical Care Medicine, Zhongshan Hospital, Fudan University, No. 180 Fenglin Road, Xuhui District, Shanghai, 200032 China

Awake prone positioning (APP) has gained prominence as a therapeutic intervention for acute respiratory distress syndrome (ARDS), particularly in COVID-19-related respiratory failure due to its proven survival benefits [1, 2]. However, the clinical applicability of APP in non-COVID-19 ARDS populations remains controversial, with patient tolerance and heterogeneous lung recruitment responses posing significant challenges [3]. To address these limitations, electromagnetic impedance tomography (EIT)—a non-invasive, radiation-free imaging modality—provides dynamic regional ventilation monitoring through real-time bedside visualization of pulmonary impedance changes [4]. We illustrate the integration of EIT-derived ventilation mapping to guide personalized positioning strategies in a non-intubated patient with moderate ARDS, demonstrating its potential to optimize alveolar recruitment while mitigating positional intolerance.

A 61-year-old female with stage IIIC lung cancer, previously treated with chemotherapy and immune checkpoint inhibitors (ICIs), developed fatal ICI-related myocarditis. Two months post-treatment, she presented with dyspnea and acute hypoxic respiratory failure (P/F ratio: 143 mmHg, ROX index: 5.6, on high-flow nasal cannula (HFNC)) due to Pneumocystis jirovecii pneumonia (PCP). However, standard awake prone positioning was contraindicated due to worsening chest tightness and dyspnea. Over three days, we continuously monitored S/F ratio, respiratory rate, and ROX index using EIT while testing various positional adjustments (Fig. 1 A–F). The “Thinker’s position” demonstrated optimal oxygenation and was maintained for approximately 6 h daily, which was her tolerance limit [5]. The patient was successfully weaned from HFNC after 12 days. Follow-up CT at day 17 showed significant inflammatory resolution, and she was discharged on day 18.

**Fig. 1:**
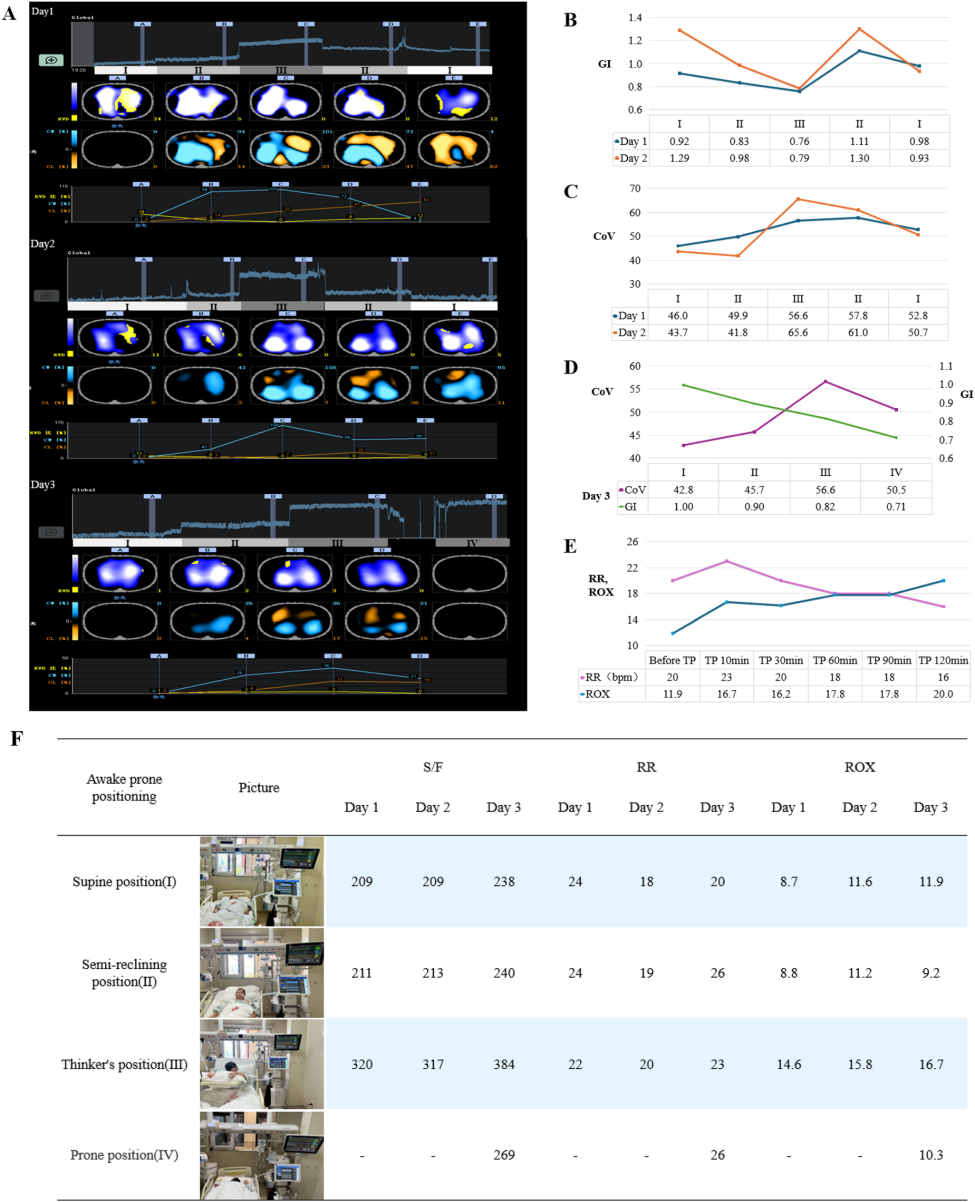
Changes in lung ventilation status and S/F, RR, and ROX of the patient in different positions under EIT monitoring. **A** shows the EIT images from the first day to the third day. The images in each panel from top to bottom are: global impedance waveforms, tidal impedance variation distribution (RVD: region ventilation delay, in yellow), difference image (CW: compliance win, in turquoise; CL: compliance loss, in orange), and data trend chart. (I), (II), (III), and (IV) in Figure A represent the supine position, semi-recumbent position, “Thinker’s position”, and prone position respectively, and each position was maintained for 10 min. **B** shows the changes in the global inhomogeneity index (GI) of the lungs in different positions monitored by EIT on the first and second days. **C** shows the changes in the ventilation center (CoV) of the lungs in different positions monitored by EIT on the first and second days. **D** shows the changes in GI and CoV of the lungs in different positions monitored by EIT on the third day. **E** shows the changes in the patient’s respiratory rate and ROX index during the 2-h maintenance of the “Thinker’s position (TP)”. **F** shows the changes in S/F, RR, and ROX of the patient in different positions from the first day to the third day

In this case, the patient demonstrated rapid oxygenation improvement following ventilation therapy in the “Thinker’s position”, representing a critical intervention in the comprehensive management of this condition. Comparative analysis of various positioning strategies revealed that both the S/F ratio and ROX index reached their optimal values in the “Thinker’s position”, with sustained improvement observed over time. EIT monitoring data indicated maximal end-expiratory lung volume (EELV) in both prone position and “Thinker’s position”. While the GI index was minimized in the prone position, followed by the “Thinker’s position”, the latter demonstrated higher CoV values. The physiological benefits of the “Thinker’s position” included increased EELV, enhanced ventilation homogeneity, and improved dorsal ventilation distribution, potentially accounting for the observed oxygenation enhancement. Although the patient attempted to resume the standard prone position on day 3, she experienced significant discomfort and requested to return to the supine position after 2 h. Due to the persistent discomfort and lack of oxygenation benefit, she declined any further attempts to adopt the standard prone position. Following the implementation of the “Thinker’s position”, no immediate improvement in respiratory rate was observed. However, a progressive reduction in respiratory rate was noted during subsequent monitoring (Fig. 1E). These findings suggest that the systematic evaluation of optimal awake prone positioning strategies, facilitated by EIT monitoring, represents a valuable approach for enhancing oxygenation parameters and optimizing clinical outcomes.

## Data Availability

The datasets analyzed during the current study are available.
