# Correction: Electrical impedance tomography-guided the optimal awake prone position in a moderate ARDS patient

**DOI:** 10.1186/s13054-025-05584-4

**Published:** 2025-08-01

**Authors:** Yongzhen Sun, Jiale Tao, Jinjun Jiang, Shujing Chen

**Affiliations:** 1 Department of Pulmonary and Critical Care Medicine, The Affiliated Hospital of ShaoXing University, Zhejiang, China; 2 Department of Pulmonary and Critical Care Medicine, Zhongshan Hospital, Fudan University, No. 180 Fenglin Road, Xuhui District, Shanghai, 200032 China

**Correction: Crit Care (2025) 29:95** 10.1186/s13054-025-05332-8

Following publication of the original article [1], the authors identified an error in Fig. 1F. Thinker’s position should be 320, however it appeared as 20. Both the incorrect and correct Fig. 1 is given hereafter.

The incorrect Fig. 1:
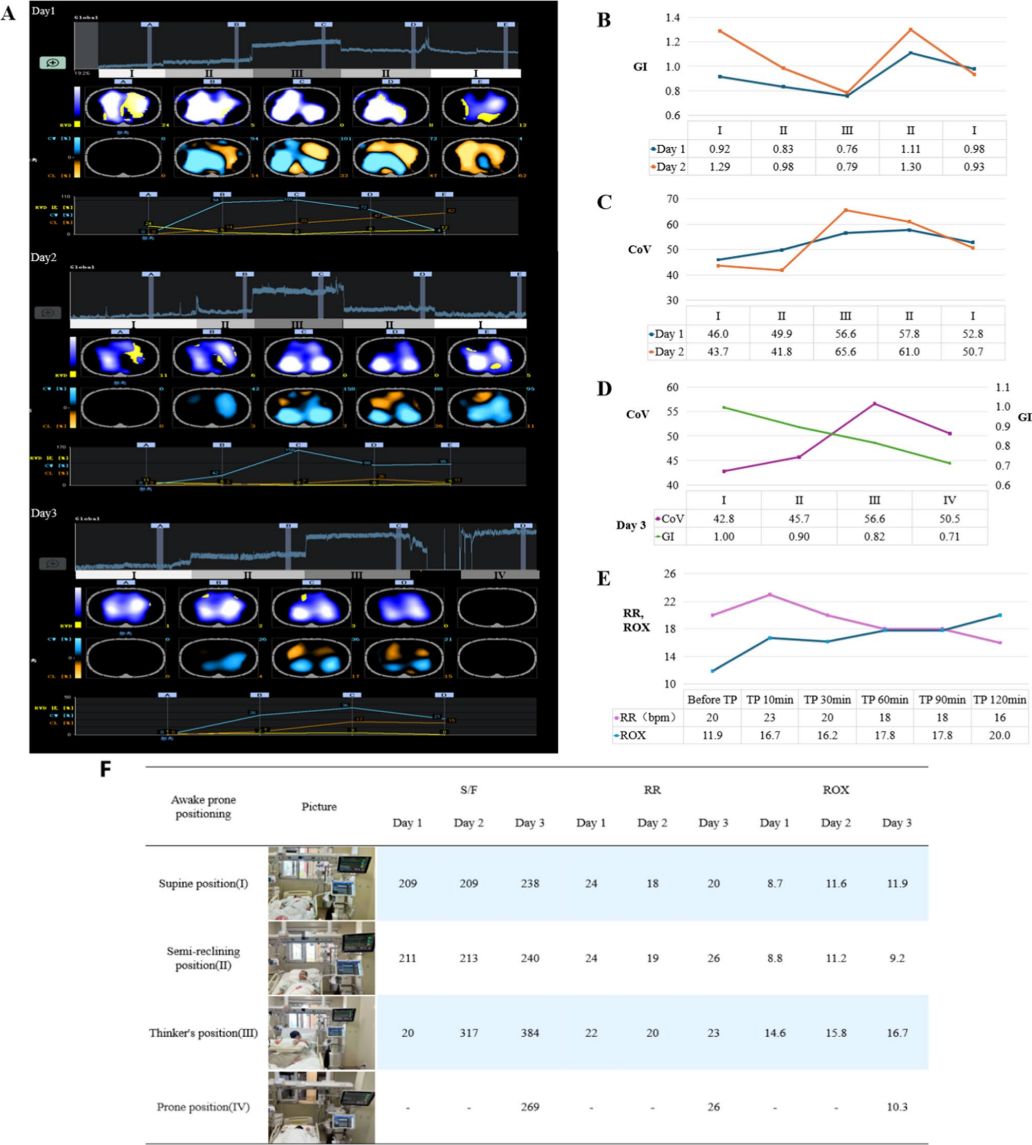


**Fig. 1** Changes in lung ventilation status and S/F, RR, and ROX of the patient in different positions under EIT monitoring. **A** shows the EIT images from the first day to the third day. The images in each panel from top to bottom are: global impedance waveforms, tidal impedance variation distribution (RVD: region ventilation delay, in yellow), difference image (CW: compliance win, in turquoise; CL: compliance loss, in orange), and data trend chart. (I), (II), (III), and (IV) in Figure A represent the supine position, semi-recumbent position, “Thinker’s position”, and prone position respectively, and each position was maintained for 10 min. **B** shows the changes in the global inhomogeneity index (GI) of the lungs in different positions monitored by EIT on the first and second days.** C** shows the changes in the ventilation center (CoV) of the lungs in different positions monitored by EIT on the first and second days.** D **shows the changes in GI and CoV of the lungs in different positions monitored by EIT on the third day. E shows the changes in the patient’s respiratory rate and ROX index during the 2-h maintenance of the“Thinker’s position (TP)”. F shows the changes in S/F, RR, and ROX of the patient in different positions from the first day to the third day

The correct Fig. 1:

**Fig. 1:**
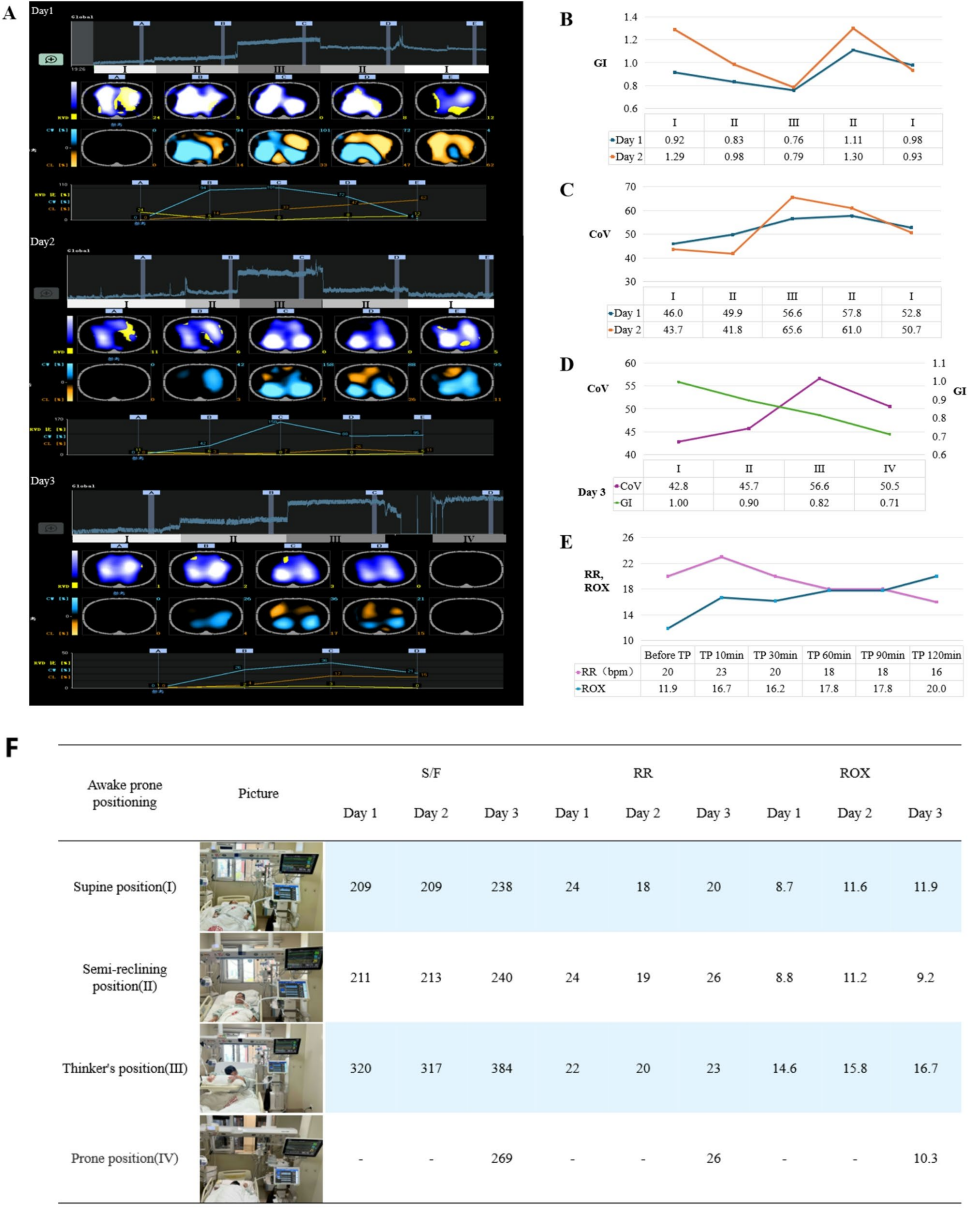
Changes in lung ventilation status and S/F, RR, and ROX of the patient in different positions under EIT monitoring. **A** shows the EIT images from the first day to the third day. The images in each panel from top to bottom are: global impedance waveforms, tidal impedance variation distribution (RVD: region ventilation delay, in yellow), difference image (CW: compliance win, in turquoise; CL: compliance loss, in orange), and data trend chart. (I), (II), (III), and (IV) in Figure A represent the supine position, semi-recumbent position, “Thinker’s position”, and prone position respectively, and each position was maintained for 10 min. **B** shows the changes in the global inhomogeneity index (GI) of the lungs in different positions monitored by EIT on the first and second days.** C** shows the changes in the ventilation center (CoV) of the lungs in different positions monitored by EIT on the first and second days.** D **shows the changes in GI and CoV of the lungs in different positions monitored by EIT on the third day. E shows the changes in the patient’s respiratory rate and ROX index during the 2-h maintenance of the“Thinker’s position (TP)”. F shows the changes in S/F, RR, and ROX of the patient in different positions from the first day to the third day

Figure 1(F) has been updated in this correction article and the original article [1] has been corrected. 
